# Timing of neonatal stoma closure: a survey of health professional perspectives and current practice

**DOI:** 10.1136/archdischild-2021-322040

**Published:** 2021-08-19

**Authors:** Jonathan Ducey, Ann M Kennedy, Louise Linsell, Kerry Woolfall, Nigel J Hall, Chris Gale, Cheryl Battersby, Gareth Penman, Marian Knight, Nick Lansdale

**Affiliations:** 1 Department of Paediatric and Neonatal Surgery, Royal Manchester Children's Hospital, Manchester, UK; 2 National Perinatal Epidemiology Unit, Nuffield Department of Population Health, University of Oxford, Oxford, UK; 3 Department of Public Health, Policy and Systems, University of Liverpool, Liverpool, UK; 4 University Surgery Unit, Faculty of Medicine, University of Southampton, Southampton, UK; 5 Faculty of Medicine, School of Public Health, Imperial College London, London, UK; 6 Newborn Intensive Care Unit, St Mary’s Hospital, Manchester, UK; 7 Faculty of Biology, Medicine and Health, The University of Manchester, Manchester, UK

**Keywords:** neonatology, growth, gastroenterology, qualitative research

## Abstract

Optimal timing for neonatal stoma closure remains unclear. In this study, we aimed to establish current practice and illustrate multidisciplinary perspectives on timing of stoma closure using an online survey sent to all 27 UK neonatal surgical units, as part of a research programme to determine the feasibility of a clinical trial comparing ‘early’ and ‘late’ stoma closure. 166 responses from all 27 units demonstrated concordance of opinion in target time for closure (6 weeks most commonly stated across scenarios), although there was a high variability in practice. A sizeable proportion (41%) of respondents use weight, rather than time, to determine when to close a neonatal stoma. Thematic analysis of free text responses identified nine key themes influencing decision-making; most related to nutrition, growth and stoma complications. These data provide an overview of current practice that is critical to informing an acceptable trial design.

## Abstract (precis)

This survey reports the perspectives of 166 respondents from 27 UK neonatal surgical centres regarding timing of neonatal stoma closure

## Introduction

Neonates undergoing emergency abdominal surgery frequently require stoma formation. Reversing (closing) stomas with a second operation is an essential part of the infant’s recovery, but evidence to inform the optimum timing of stoma closure is limited and conflicting.^1–4^ The Timing of Stoma Closure in Neonates (ToSCiN) study will use mixed methods to determine the feasibility of a clinical trial comparing ‘early’ and ‘late’ stoma closure in neonates. In this part, we undertook a survey of neonatal surgical professionals in the UK to establish current practice for stoma closure in neonates and explore their perspectives to inform future trial design.

## Methods

An online survey was developed which focused on three domains (online supplemental appendix 1):

10.1136/fetalneonatal-2021-322040.supp1 Supplementary data

1. The clinical role of the respondent and structure of their neonatal surgical institution.
2. Current practice for timing of stoma closure and clinical considerations for expediting or delaying surgery.
3. Focused questions on preferred practice across four clinical scenarios: preterm and term infants with congenital or acquired gut pathologies.

The survey was distributed using LimeSurvey to consultant neonatologists, consultant paediatric surgeons, neonatal dieticians and specialist neonatal surgical nurses in all 27 UK units caring for surgical neonates. We also disseminated the survey via national organisations (British Association of Paediatric Surgeons and British Association of Perinatal Medicine) and personal contacts of the study team. Our target response was ≥2 surgeons and ≥2 neonatologists from each centre, while ensuring sampling variance (eg, geographical area and healthcare professional type). Finally, we used snowball sampling whereby invitees were encouraged to cascade the survey to help maximise responses. Reminder emails were sent to potential participants to encourage completion and the survey was open for 3 months.

Survey data were described using counts and percentages for categorical variables and median (IQR) or mode for continuous variables. Qualitative free text responses were indexed and analysed thematically.^5^

## Results

A total of 166 professionals across all 27 UK neonatal surgical centres completed the survey, with 87 (52%) responses from surgeons, 66 (40%) neonatologists, 8 (5%) specialist nurses and 5 (3%) dieticians. All UK neonatal surgical centres responded: ≥2 surgeons responded from 24/27 centres and ≥2 neonatologists from 24/27.

Without defining ‘early’ or ‘later’, 78/166 (47%) respondents generally considered themselves proponents of ‘early’ stoma closure, 47/166 (28%) proponents of ‘later’ stoma closure and 41/166 (25%) unsure.

### Achieving a predefined time interval prior to stoma closure

Attitudes towards timing of stoma closure were sought for each of four clinical scenarios (table 1). The target time to closure was most commonly 6 weeks, although there was variability between scenarios; more respondents favoured a longer time interval between stoma formation and closure for premature infants. Common free text explanations for a longer interval suggested that greater morbidity and instability would be expected in premature or very-low-birthweight infants. Inflammatory pathologies (ie, bowel perforation, peritonitis and necrotising enterocolitis) were often considered to be justifications to extend the interval between procedures to allow evolution of gut sequelae and abdominal quiescence, along with resolution of the catabolic phase of recovery (table 2). While 70%–76% of respondents preferred stoma closure prior to discharge in preterm infants and a term infant with a jejunostomy, only 46% would prefer this approach in the term infant with an ileostomy. In all scenarios, a sizeable proportion (29%–37%) of respondents indicated they were unsure when would be best to close the stoma.

**Table 1:** : Summary of responses to questions around clinical scenarios

	Scenario 1	Scenario 2	Scenario 3	Scenario 4
	*‘A premature infant born at 26 weeks’ gestation (birth weight 800 g) deteriorates clinically on day 3 of life. An isolated perforation of the distal small bowel (ileum) is found at laparotomy and a stoma and mucous fistula are formed at this level.’*	*‘A premature infant born at 26 weeks’ gestation (birth weight 800 g) develops clinical signs of NEC at 4 weeks of age. A laparotomy confirms diffuse small bowel involvement and 50 cm of bowel is resected. A stoma and mucous fistula are formed at the level of the mid-jejunum.’*	*‘A term infant is born with signs of distal bowel obstruction and a failure to pass meconium. “Simple” meconium ileus and a micro-colon are found at laparotomy. A stoma and mucous fistula are formed in the mid-ileum.’*	*‘A term infant is born with signs of proximal bowel obstruction and a failure to pass meconium. At laparotomy, a jejunal atresia is found. A stoma and mucous fistula are formed at the site of the atresia (mid-jejunum).’*
Target stoma closure time	n=118/166 (71%) weeks specified: median (IQR)=8 (6, 12), mode=6 n=48/166 (29%) unsure	n=110/166 (66%) weeks specified: median (IQR)=8 (6, 10), mode=6 n=56/166 (34%) unsure	n=105/166 (63%) weeks specified: median (IQR)=4 (6, 8), mode=6 n=61/166 (37%) unsure	n=104/166 (63%) weeks specified: median (IQR)=6 (4, 6), mode=6 n=62/166 (37%) unsure
Preference for closure in relation to discharge	Before: 117 (70%) After: 25 (15%) Unsure: 24 (14%)	Before: 126 (76%) After: 15 (9%) Unsure: 25 (15%)	Before: 77 (46%) After: 53 (32%) Unsure: 36 (22%)	Before: 120 (72%) After: 26 (16%) Unsure: 20 (12%)
Earliest experience of closure in comparable scenario	n=153/166 (92%) weeks specified: median (IQR)=6 (4, 6), mode=6	n=153/166 (92%) weeks specified: median (IQR)=6 (4, 8), mode=6	n=135/166 (81%) weeks specified: median (IQR)=4 (4, 6), mode=4	n=125/166 (75%) weeks specified: median (IQR)=4 (3, 6), mode=4
Latest experience of closure in comparable scenario	n=154/166 (93%) weeks specified: median (IQR)=20 (12, 30), mode=12	n=143/166 (86%) weeks specified: median (IQR)=20 (12, 26), mode=12	n=126/166 (76%) weeks specified: median (IQR)=16 (12, 24), mode=12	n=115/166 (69%) weeks specified: median (IQR)=12 (8, 16), mode=12

NEC, necrotising enterocolitis.

**Table 2:** : Core themes identified from qualitative analysis of free text responses

Theme	Subtheme (number of respondents who mentioned this theme)	Example free text responses (respondent role)
Factors supporting expediting stoma closure	Growth failure and PN dependence (including liver disease) (127)	*‘If there are growth issues, PN [parenteral nutrition] requirement or stoma complications, I would aim for an early closure.’* (Respondent 146, Paediatric surgeon)
	High-output (or proximal) stoma (112)	*‘If a baby is failing to thrive, high output stoma losses and TPN [total parenteral nutrition] dependent then we would close sooner rather than later and certainly before discharge.’* (Respondent 150, Neonatologist) *‘Usually, with high-output stomas, early closure is required to avoid growth failure. However, a stably growing infant with successful pro-cycling of stoma outputs can have delayed closure.’* (Respondent 11, Neonatologist)
	Peristomal issues (eg, skin breakdown, prolapse, granulation) (26)	
	Social issues (18)	*‘…if the baby had difficult social situation and the risk of a stoma at home and picking up issues high [risk] then would advocate for early closure.’* (Respondent 154, Neonatal surgical specialist nurse) *‘Timing of stoma closure is multifactorial—depends on stoma care, stoma complications, level of stoma, success of recycling, distance family are from home, social circumstances, tolerance of feed, IV [intravenous] access, parent’s wishes, other pathology, and many other factors that is, it is individualised to each child and family.’* (Respondent 22, Paediatric surgeon)
	Vascular access (7)	
Factors supporting delaying planned stoma closure	Thriving with stoma and enterally autonomous (including successful recycling) (109)	*‘I am not sure if the timing of stoma closure is my main concern as a neonatologist. My main concern is time to full feeds, and growth rate. Stoma closure is secondary, and I am happy to consider discharge home with a stoma. In fact, I would prefer to discharge this baby home with a stoma than prolong hospital stay to achieve closure before discharge home.’* (Respondent 165, Neonatologist) *‘Ideally if patient is well with good stoma management and recycling and gaining weight then I would wait until a few months of age.’* (Respondent 59, Paediatric surgeon)
	Comorbidities (not optimised for surgery and/or anaesthetic) (56)	*‘This baby is more likely to have co-morbidities which will influence surgical and anaesthetic risk, especially CLD [chronic lung disease]…Some of these babies get closed many months post-discharge if they are complicated.’* (Respondent 28, Neonatologist)
	Underlying gut pathology/surgical technical concerns (35)	*‘Ideally, I would wait for 6 weeks after NEC [necrotising enterocolitis], to allow for maturation and identification of post-NEC strictures, which may have bearing on success of closure …I would move to close sooner if MDT [multidisciplinary team] discussion agreed best for baby; again, other comorbidities have a bearing on timing of closure.’* (Respondent 34, Paediatric surgeon)
	Difficulty accessing theatre lists (including COVID-19 limitations) (14)	*‘One of our main confounding issues at the present time is timely access to theatre lists. Our capacity was diminished pre COVID and worse now. The 1 year wait for stoma closure was certainly not through choice but reflects this problem.’* (Respondent 144, Paediatric surgeon)

### Reasons for delaying stoma closure

Forty per cent (60/150) of respondents reported they would delay stoma closure due to ‘Invasive ventilation (clinically stable and low/moderate support)’, 29% (44/150) due to ‘non-invasive respiratory support (clinically stable, BIPAP, CPAP, high flow oxygen)’ and 58% (80/138) due to ‘Steroids within the last week’.

### Achieving a defined weight threshold prior to stoma closure

Weight was a recurrent theme in both free text responses and closed questions on preferred timing of stoma closure. Just under half (54/132, 41%) of the respondents would delay stoma closure until an infant had reached a predefined weight, although the weight threshold varied: median (IQR) 2000 g (1625–2500 g); mode 2500 g.

### Reasons for expediting stoma closure

The large majority of respondents indicated they would bring stoma closure forward if there were problems with an infant’s growth, nutrition and stoma. Ninety-six per cent (158/164) reported they would expedite closure due to ‘Concern about poor growth due to stoma’, 97% (157/162) due to ‘Parenteral nutrition (PN) issues (eg, liver disease or recurrent line sepsis)’, 93% (152/163) due to ‘Inability to advance enteral feeds due to stoma outputs’ and 95% (154/162) due to ‘Difficulties with managing the stoma (eg, leaking bags, prolapse)’. Recycling of stoma effluent was a variable practice among respondents: ‘Routine’=41/164 (25%), ‘Sometimes’=68/164 (41%), ‘Rarely’=42/164 (26%) and ‘Never’=13/164 (8%). Fifty-two per cent (73/140) reported they would expedite stoma closure due to ‘Inability to recycle stoma effluent distally’.

### Qualitative analysis of free text responses

For each clinical scenario, respondents were asked for further comments and considerations on timing of stoma closure. Through thematic analysis of 355 free text responses, nine key themes were identified as factors influencing decision-making. Those supporting expediting stoma closure included: growth failure and PN dependence, high-output stoma, peristomal problems and vascular access. Factors supporting delaying stoma closure included: thriving with stoma, comorbidities (not optimised for surgery), underlying gut pathology/surgical technical concerns and difficulty accessing theatre lists (table 2).

## Discussion

This large study of practice and practitioner views illustrates the sometimes conflicting clinical variables that impact the timing of stoma closure. While there appears to be some concordance of opinion about an initial target time for closure (6-week interval most common for all scenarios), there remains a high degree of variability, with intervals of 12 weeks or more frequently advocated. This study also demonstrates that a sizeable proportion (41%) of respondents use weight, rather than time, to determine when to close a neonatal stoma. Furthermore, one in three respondents expressed uncertainty about timing of stoma closure, perhaps highlighting the lack of a clear evidence base in this area but also the difficulty in committing to a time when faced with sometimes conflicting and changing clinical parameters in an already heterogenous population.

While often life-saving, neonatal stomas can lead to significant challenges, with morbidity from fluid and electrolyte imbalances, peristomal complications, consequences of PN and the need for central vascular access.^1 2^ Our survey reflects these concerns and highlights nine key factors that affect timing of closure, with the majority relating to nutrition and growth. Perhaps unsurprisingly, we found that a respondent’s clinical role altered their responses (eg surgeons more often quoted technical factors and neonatologists medical factors as reasons for delaying or expediting surgery). However, the above-mentioned key factors were common to responses from all professions.

A key strength of our survey is its wide coverage with multidisciplinary responses from all UK neonatal surgical units. As with most surveys of practice, a limitation is that respondents reported what they believe their practice to be, rather than providing data on actual clinical cases. We attempted to mitigate against this through provision of real-world clinical scenarios and will capture observational data about practice in future work within the ToSCiN study.

A clinical trial comparing ‘early’ versus ‘late’ stoma closure would provide high-quality evidence on which to base decision-making. This work provides an overview of current practice that will be critical to informing acceptable trial design.

Future work will further determine trial feasibility based around real-world cases using quantitative and qualitative methods. This will include assessing eligibility in a group of infants with a range of clinical characteristics (and how this may change over time) and whether a trial of early versus late closure would be acceptable to parents and clinicians caring for these infants.
