# Supplementary material for: Timing of neonatal stoma closure: a survey of health professional perspectives and current practice
Source: Arch Dis Child Fetal Neonatal Ed. 2021 Aug 19;107(4):448–50. doi: 10.1136/archdischild-2021-322040 (PMC9209674; doi:10.1136/archdischild-2021-322040)
Supplement: Supplementary data [file fetalneonatal-2021-322040supp001.pdf]

Timing of Stoma Closure in Neonates (ToSCiN)

ToSCiN aims to answer the question, ‘is it feasible to conduct a clinical trial comparing ‘early’ vs ‘late’ stoma closure in neonates?’

This survey aims to determine the viewpoints of UK clinicians and allied health professionals who regularly care for infants with stomas.

We would be very grateful if you could complete the survey: we will provide feedback of the results at the end of the ToSCiN study.

There are 50 questions in this survey.

Background Information

Your role *

Please choose **only one** of the following:

- ◯ Consultant Paediatric Surgeon
- ◯ Consultant Neonatologist/Paediatrician (regularly cares for infants with stomas)
- ◯ Neonatal Surgical Specialist Nurse
- ◯ Dietician
- ◯ Other

Ducey J, *et al. Arch Dis Child Fetal Neonatal Ed* 2022; 107:448–450. doi: 10.1136/archdischild-2021-322040

Description of the type of unit you work in: *

Only answer this question if the following conditions are met:
Answer was 'Dietician' *or* 'Neonatal Surgical Specialist Nurse' *or* 'Consultant Neonatologist/Paediatrician (regularly cares for infants with stomas)' *or* 'Consultant Paediatric Surgeon' at question '1 [A01]' (Your role)

Please choose **only one** of the following:

- ◯ Surgical centre with co-located Neonatal Intensive Care Unit
- ◯ Neonatal Intensive Care Unit with partner Neonatal Surgical Centre at separate site*
- ◯ Neonatal Surgical Centre with partner Neonatal Intensive Care at separate site*
- ◯ Other

* ‘Separate site’ defined as a requirement for external transfer of an infant from NICU to operating theatre for surgery

Thank you for your interest but this survey is specifically seeking the viewpoints of those with the above roles

Only answer this question if the following conditions are met:
Answer was 'Other' at question '1 [A01]' (Your role)

Thank you for your interest but this survey is specifically seeking the viewpoints of those working in the above types of units

Only answer this question if the following conditions are met:
Answer was 'Other' at question '2 [A02]' (Description of the type of unit you work in:)

Introduction

Accepting there is no consensus/definition for ‘early’ or ‘late', would you consider yourself a proponent of ‘early’ or ‘later’ stoma closure in infants who have had a stoma formed in the neonatal period? *

Only answer this question if the following conditions are met:
Answer was NOT 'Other' at question '1 [A01]' (Your role) *and* Answer was NOT 'Other' at question '2 [A02]' (Description of the type of unit you work in:)

Please choose **only one** of the following:

- ◯ Early
- ◯ Later
- ◯ Unsure

Does your unit recycle stoma output through the mucous fistula?

Only answer this question if the following conditions are met:
Answer was NOT 'Other' at question '1 [A01]' (Your role) *and* Answer was NOT 'Other' at question '2 [A02]' (Description of the type of unit you work in:)

Please choose **only one** of the following:

- ◯ Routine
- ◯ Sometimes
- ◯ Rarely
- ◯ Never

Scenarios

We will now describe 2 preterm and 2 term infant scenarios. Please indicate, in terms of your current practice, how you feel it would be best to manage each case.

Only answer this question if the following conditions are met:
Answer was NOT 'Other' at question '1 [A01]' (Your role) *and* Answer was NOT 'Other' at question '2 [A02]' (Description of the type of unit you work in:)

Ducey J, *et al. Arch Dis Child Fetal Neonatal Ed* 2022; 107:448–450. doi: 10.1136/archdischild-2021-322040

Very preterm infant scenarios: Scenario 1

A premature infant born at 26 weeks gestation (birth weight 800g) deteriorates clinically on day 3 of life. An isolated perforation of the distal small bowel (ileum) is found at laparotomy and a stoma and mucous fistula are formed at this level.

At this point (immediately after stoma formation), after how many weeks do you think the stoma should be aimed to be closed? *

Only answer this question if the following conditions are met:
Answer was NOT 'Other' at question '1 [A01]' (Your role) *and* Answer was NOT 'Other' at question '2 [A02]' (Description of the type of unit you work in:)

Please choose **only one** of the following:

- ◯ Number of weeks (enter below)
- ◯ Unsure

Please enter number of weeks: *

Only answer this question if the following conditions are met:
Answer was 'Number of weeks (enter below)' at question '8 [Sc101]' (At this point (immediately after stoma formation), after how many weeks do you think the stoma should be aimed to be closed?)

❗ Only an integer value may be entered in this field.
Please write your answer here:

[ ]

Would you normally want the stoma closed before or after discharge home? *

Only answer this question if the following conditions are met:
Answer was NOT 'Other' at question '1 [A01]' (Your role) *and* Answer was NOT 'Other' at question '2 [A02]' (Description of the type of unit you work in:)

Please choose **only one** of the following:

- ◯ Before
- ◯ After
- ◯ Unsure

In your experience, what's the earliest you have seen stoma closure performed in a baby like this?

Only answer this question if the following conditions are met:
Answer was NOT 'Other' at question '1 [A01]' (Your role) *and* Answer was NOT 'Other' at question '2 [A02]' (Description of the type of unit you work in:)

Please write your answer here:

weeks post formation

In your experience, what's the latest you have seen stoma closure performed in a baby like this?

Only answer this question if the following conditions are met:
Answer was NOT 'Other' at question '1 [A01]' (Your role) *and* Answer was NOT 'Other' at question '2 [A02]' (Description of the type of unit you work in:)

Please write your answer here:

weeks post formation

Any further comments:

Only answer this question if the following conditions are met:
Answer was NOT 'Other' at question '1 [A01]' (Your role) *and* Answer was NOT 'Other' at question '2 [A02]' (Description of the type of unit you work in:)

Please write your answer here:

Very preterm infant scenarios: Scenario 2

A premature infant born at 26 weeks gestation (birth weight 800g) develops clinical signs of NEC at 4 weeks of age. A laparotomy confirms diffuse small bowel involvement and 50cm of bowel is resected. A stoma and mucous fistula are formed at the level of the mid-jejunum.

At this point (immediately after stoma formation), after how many weeks do you think the stoma should be aimed to be closed? *

Only answer this question if the following conditions are met:
((A01.NAOK (/index.php/admin/questions/sa/view/surveyid/675993/gid/7/qid/167) != "A5") and (A02.NAOK (/index.php/admin/questions/sa/view/surveyid/675993/gid/7/qid/168) != "A4"))

Please choose **only one** of the following:

- ◯ Number of weeks (enter below)
- ◯ Unsure

Please enter number of weeks: *

Only answer this question if the following conditions are met:
Answer was 'Number of weeks (enter below)' at question '14 [Sc201]' (At this point (immediately after stoma formation), after how many weeks do you think the stoma should be aimed to be closed?)

Only an integer value may be entered in this field.
Please write your answer here:

Would you normally want the stoma closed before or after discharge home? *

Only answer this question if the following conditions are met:
((A01.NAOK (/index.php/admin/questions/sa/view/surveyid/675993/gid/7/qid/167) != "A5") and (A02.NAOK (/index.php/admin/questions/sa/view/surveyid/675993/gid/7/qid/168) != "A4"))

Please choose **only one** of the following:

- Before
- After
- Unsure

In your experience, what's the earliest you have seen stoma closure performed in a baby like this?

Only answer this question if the following conditions are met:
((A01.NAOK (/index.php/admin/questions/sa/view/surveyid/675993/gid/7/qid/167) != "A5") and (A02.NAOK (/index.php/admin/questions/sa/view/surveyid/675993/gid/7/qid/168) != "A4"))

Please write your answer here:

weeks post formation

In your experience, what's the latest you have seen stoma closure performed in a baby like this?

Only answer this question if the following conditions are met:
((A01.NAOK (/index.php/admin/questions/sa/view/surveyid/675993/gid/7/qid/167) != "A5") and (A02.NAOK (/index.php/admin/questions/sa/view/surveyid/675993/gid/7/qid/168) != "A4"))

Please write your answer here:

weeks post formation

Any further comments:

Only answer this question if the following conditions are met:
((A01.NAOK (/index.php/admin/questions/sa/view/surveyid/675993/gid/7/qid/167) != "A5") and (A02.NAOK (/index.php/admin/questions/sa/view/surveyid/675993/gid/7/qid/168) != "A4"))

Please write your answer here:

Scenarios for term infants: Scenario 1

A term infant is born with signs of distal bowel obstruction and a failure to pass meconium. 'Simple' meconium ileus and a micro-colon are found at laparotomy. A stoma and mucous fistula are formed in the mid-ileum.

At this point (immediately after stoma formation), after how many weeks do you think the stoma should be aimed to be closed? *

Only answer this question if the following conditions are met:
((A01.NAOK (/index.php/admin/questions/sa/view/surveyid/675993/gid/7/qid/167) != "A5") and (A02.NAOK (/index.php/admin/questions/sa/view/surveyid/675993/gid/7/qid/168) != "A4"))

Please choose **only one** of the following:

- ◯ Number of weeks (enter below)
- ◯ Unsure

Please enter number of weeks: *

Only answer this question if the following conditions are met:
Answer was 'Number of weeks (enter below)' at question '20 [Sc301]' (At this point (immediately after stoma formation), after how many weeks do you think the stoma should be aimed to be closed?)

❗ Only an integer value may be entered in this field.
Please write your answer here:

Would you normally want the stoma closed before or after discharge home? *

Only answer this question if the following conditions are met:
((A01.NAOK (/index.php/admin/questions/sa/view/surveyid/675993/gid/7/qid/167) != "A5") and (A02.NAOK (/index.php/admin/questions/sa/view/surveyid/675993/gid/7/qid/168) != "A4"))

Please choose **only one** of the following:

- ◯ Before
- ◯ After
- ◯ Unsure

In your experience, what's the earliest you have seen stoma closure performed in a baby like this?

Only answer this question if the following conditions are met:
((A01.NAOK (/index.php/admin/questions/sa/view/surveyid/675993/gid/7/qid/167) != "A5") and (A02.NAOK (/index.php/admin/questions/sa/view/surveyid/675993/gid/7/qid/168) != "A4"))

Please write your answer here:

weeks post formation

In your experience, what's the latest you have seen stoma closure performed in a baby like this?

Only answer this question if the following conditions are met:
((A01.NAOK (/index.php/admin/questions/sa/view/surveyid/675993/gid/7/qid/167) != "A5") and (A02.NAOK (/index.php/admin/questions/sa/view/surveyid/675993/gid/7/qid/168) != "A4"))

Please write your answer here:

weeks post formation

Any further comments:

Only answer this question if the following conditions are met:
((A01.NAOK (/index.php/admin/questions/sa/view/surveyid/675993/gid/7/qid/167) != "A5") and (A02.NAOK (/index.php/admin/questions/sa/view/surveyid/675993/gid/7/qid/168) != "A4"))

Please write your answer here:

Ducey J, *et al. Arch Dis Child Fetal Neonatal Ed* 2022; 107:448–450. doi: 10.1136/archdischild-2021-322040

Scenarios for term infants: Scenario 2

A term infant is born with signs of proximal bowel obstruction and a failure to pass meconium. At laparotomy, a jejunal atresia is found. A stoma and mucous fistula are formed at the site of the atresia (mid-jejunum).

At this point (immediately after stoma formation), after how many weeks do you think the stoma should be aimed to be closed? *

Only answer this question if the following conditions are met:
((A01.NAOK (/index.php/admin/questions/sa/view/surveyid/675993/gid/7/qid/167) != "A5") and (A02.NAOK (/index.php/admin/questions/sa/view/surveyid/675993/gid/7/qid/168) != "A4"))

Please choose **only one** of the following:

- ◯ Number of weeks (enter below)
- ◯ Unsure

Please enter number of weeks: *

Only answer this question if the following conditions are met:
Answer was 'Number of weeks (enter below)' at question '26 [Sc401]' (At this point (immediately after stoma formation), after how many weeks do you think the stoma should be aimed to be closed?)

❗ Only an integer value may be entered in this field.
Please write your answer here:

Ducey J, *et al. Arch Dis Child Fetal Neonatal Ed* 2022; 107:448–450. doi: 10.1136/archdischild-2021-322040

Would you normally want the stoma closed before or after discharge home? *

Only answer this question if the following conditions are met:
((A01.NAOK (/index.php/admin/questions/sa/view/surveyid/675993/gid/7/qid/167) != "A5") and (A02.NAOK (/index.php/admin/questions/sa/view/surveyid/675993/gid/7/qid/168) != "A4"))

Please choose **only one** of the following:

- ◯ Before
- ◯ After
- ◯ Unsure

In your experience, what's the earliest you have seen stoma closure performed in a baby like this?

Only answer this question if the following conditions are met:
((A01.NAOK (/index.php/admin/questions/sa/view/surveyid/675993/gid/7/qid/167) != "A5") and (A02.NAOK (/index.php/admin/questions/sa/view/surveyid/675993/gid/7/qid/168) != "A4"))

Please write your answer here:

weeks post formation

In your experience, what's the latest you have seen stoma closure performed in a baby like this?

Only answer this question if the following conditions are met:
((A01.NAOK (/index.php/admin/questions/sa/view/surveyid/675993/gid/7/qid/167) != "A5") and (A02.NAOK (/index.php/admin/questions/sa/view/surveyid/675993/gid/7/qid/168) != "A4"))

Please write your answer here:

weeks post formation

Any further comments:

Only answer this question if the following conditions are met:
((A01.NAOK (/index.php/admin/questions/sa/view/surveyid/675993/gid/7/qid/167) != "A5") and (A02.NAOK (/index.php/admin/questions/sa/view/surveyid/675993/gid/7/qid/168) != "A4"))

Please write your answer here:

Current Practice

Which of these factors would make you delay a planned stoma closure?

Only answer this question if the following conditions are met:
Answer was NOT 'Other' at question '1 [A01]' (Your role) *and* Answer was NOT 'Other' at question '2 [A02]' (Description of the type of unit you work in:)

Please choose the appropriate response for each item:

	**Delay**	**Go ahead**
**Invasively ventilated (clinically stable and low/moderate support)**	○	○
**Non-invasive respiratory support (clinically stable BIPAP, CPAP, high flow)**	○	○
**Weight less than a specific threshold**	○	○
**Steroids within last week**	○	○

Please specify weight threshold:

Only answer this question if the following conditions are met:
Answer was 'Delay' at question '32 [C01]' (Which of these factors would make you delay a planned stoma closure? (Weight less than a specific threshold))

Please choose **only one** of the following:

- ◯ 500g
- ◯ 1,000g
- ◯ 1,500g
- ◯ 2,000g
- ◯ 2,500g
- ◯ 3,000g
- ◯ 3,500g
- ◯ 4,000g
- ◯ 4,500g
- ◯ 5,000g

Where 'delay' has been selected for 'weight less than a specific threshold' in the question above.

Ducey J, *et al. Arch Dis Child Fetal Neonatal Ed* 2022; 107:448–450. doi: 10.1136/archdischild-2021-322040

Which of these factors would make you expedite a planned stoma closure that was still some weeks away?

Only answer this question if the following conditions are met:
Answer was NOT 'Other' at question '1 [A01]' (Your role) *and* Answer was NOT 'Other' at question '2 [A02]' (Description of the type of unit you work in:)

Please choose the appropriate response for each item:

	**Expedite**	**Unchanged**
**Unable to recycle stoma distally effluent (down mucous fistula)**	○	○
**Concern about poor growth due to stoma**	○	○
**Parenteral nutrition issues e.g. liver disease or recurrent line sepsis**	○	○
**Inability to advance enteral feeds due to stoma outputs**	○	○
**Difficulties with managing the stoma e.g. leaking bags, prolapse, stricture, skin complications**	○	○

Are there any other factors you feel are important when deciding when to close the stoma of these infants or whether you would be willing to randomise to a trial?

Only answer this question if the following conditions are met:
Answer was NOT 'Other' at question '1 [A01]' (Your role) *and* Answer was NOT 'Other' at question '2 [A02]' (Description of the type of unit you work in:)

Please write your answer here:

Perspectives on Clinical Trial Design

When answering the following questions, please consider how any future clinical trial would be most useful to you in terms of helping decision-making in your clinical practice.

Population

1. What groups of infants should be included in a trial? *

Only answer this question if the following conditions are met:
Answer was NOT 'Other' at question '1 [A01]' (Your role) *and* Answer was NOT 'Other' at question '2 [A02]' (Description of the type of unit you work in:)

Please choose the appropriate response for each item:

	Yes	No	Unsure
**Premature infants with spontaneous intestinal perforation (SIP)**	○	○	○
**Premature infants with NEC**	○	○	○
**Term infants with emergency stoma formation for bowel obstruction (e.g. atresias, meconium ileus, complicated gastroschisis or malrotation/volvulus)**	○	○	○
**Other (if yes, enter details below)**	○	○	○

We are not planning on including infants with anorectal malformations and Hirschsprung's disease, as stoma closures in these infants are usually part of a planned treatment pathway

If you have selected 'other' above, please describe here: *

Only answer this question if the following conditions are met:
Answer was 'Yes' at question '36 [E01]' (Population 1. What groups of infants should be included in a trial? (Other (if yes, enter details below)))

Please write your answer here:

Ducey J, *et al. Arch Dis Child Fetal Neonatal Ed* 2022; 107:448–450. doi: 10.1136/archdischild-2021-322040

2. Are there any babies that you don’t think should be included in a trial?

Only answer this question if the following conditions are met:
Answer was NOT 'Other' at question '1 [A01]' (Your role) *and* Answer was NOT 'Other' at question '2 [A02]' (Description of the type of unit you work in:)

Please write your answer here:

Intervention

3. What do you think the ‘early’ time-point should be in a trial of early vs. late stoma closure in neonates? *

Only answer this question if the following conditions are met:
Answer was NOT 'Other' at question '1 [A01]' (Your role) *and* Answer was NOT 'Other' at question '2 [A02]' (Description of the type of unit you work in:)

❗ If you choose 'Completed weeks after stoma formation:' please also specify your choice in the accompanying text field.
❗ Only numbers may be entered in 'Completed weeks after stoma formation:' accompanying text field.
Please choose **only one** of the following:

- ◯ Completed weeks after stoma formation (enter number of weeks below)
- ◯ Unsure

Please enter number of weeks: *

Only answer this question if the following conditions are met:
Answer was 'Completed weeks after stoma formation (enter number of weeks below)' at question '39 [E03]' (Intervention 3. What do you think the ‘early’ time-point should be in a trial of early vs. late stoma closure in neonates?)

Please write your answer here:

Comparison

4. What do you think the ‘later’ comparison should be in such a trial? *

Only answer this question if the following conditions are met:
Answer was NOT 'Other' at question '1 [A01]' (Your role) *and* Answer was NOT 'Other' at question '2 [A02]' (Description of the type of unit you work in:)

❗ If you choose 'Completed weeks after stoma formation:' please also specify your choice in the accompanying text field.
❗ Only numbers may be entered in 'Completed weeks after stoma formation:' accompanying text field.
Please choose **only one** of the following:

- ◯ Completed weeks after stoma formation (enter number of weeks below)
- ◯ Unsure

Please enter number of weeks: *

Only answer this question if the following conditions are met:
Answer was 'Completed weeks after stoma formation (enter number of weeks below)' at question '41 [E04]' (Comparison 4. What do you think the ‘later’ comparison should be in such a trial?)

Please write your answer here:

Ducey J, *et al. Arch Dis Child Fetal Neonatal Ed* 2022; 107:448–450. doi: 10.1136/archdischild-2021-322040

Outcomes

5. A core outcome set for neonatology has been identified to help standardise outcome selection in clinical trials and ensure these are relevant to those most affected by neonatal care (https://fn.bmj.com/content/105/4/425 (https://fn.bmj.com/content/105/4/425)). We would envisage these outcomes would be included in any trial of stoma closure.

In addition, there are likely to be study-specific outcomes that it are important to include. Please score how important you feel these potential trial outcome measures are.

Only answer this question if the following conditions are met:
Answer was NOT 'Other' at question '1 [A01]' (Your role) *and* Answer was NOT 'Other' at question '2 [A02]' (Description of the type of unit you work in:)

Please choose the appropriate response for each item:

	Not important	Little importance	Important	Very important	Extremely important
**Length of hospital stay**	◯	◯	◯	◯	◯
**Duration of parenteral nutrition (PN)**	◯	◯	◯	◯	◯
**Time to full enteral feeds**	◯	◯	◯	◯	◯
**Growth**	◯	◯	◯	◯	◯
**Complications of surgery e.g. anastomotic leak or stricture**	◯	◯	◯	◯	◯
**Days of invasive ventilation post-operatively**	◯	◯	◯	◯	◯
**Parental experience**	◯	◯	◯	◯	◯
**Please indicate any others you think are important (describe below)**	◯	◯	◯	◯	◯

Ducey J, *et al. Arch Dis Child Fetal Neonatal Ed* 2022; 107:448–450. doi: 10.1136/archdischild-2021-322040

For 'any others' selected above, please describe these outcomes here: *

Only answer this question if the following conditions are met:
Answer was NOT at question '43 [E06]' (Outcomes 5. A core outcome set for neonatology has been identified to help standardise outcome selection in clinical trials and ensure these are relevant to those most affected by neonatal care (https://fn.bmj.com/content/105/4/425). We would envisage these outcomes would be included in any trial of stoma closure. In addition, there are likely to be study-specific outcomes that it are important to include. Please score how important you feel these potential trial outcome measures are. (Please indicate any others you think are important (describe below)))

Please write your answer here:

Ducey J, *et al. Arch Dis Child Fetal Neonatal Ed* 2022; 107:448–450. doi: 10.1136/archdischild-2021-322040

6. What should be the primary outcome of any trial of early vs. late stoma closure?

Only answer this question if the following conditions are met:
((A01.NAOK (/index.php/admin/questions/sa/view/surveyid/675993/gid/7/qid/167) != "A5") and (A02.NAOK (/index.php/admin/questions/sa/view/surveyid/675993/gid/7/qid/168) != "A4"))

❗ Choose one of the following answers
Please choose **only one** of the following:

- ◯ Length of hospital stay
- ◯ Duration of parenteral nutrition (PN)
- ◯ Time to full enteral feeds
- ◯ Growth
- ◯ Complications of surgery e.g. anastomotic leak or stricture
- ◯ Days of invasive ventilation post-operatively
- ◯ Parental experience
- ◯ Survival
- ◯ Sepsis
- ◯ Necrotising enterocolitis
- ◯ Brain injury on imaging
- ◯ Retinopathy of prematurity (preterm only)
- ◯ General gross motor ability
- ◯ General cognitive ability
- ◯ Quality of life
- ◯ Adverse events
- ◯ Visual impairment or blindness
- ◯ Hearing impairment or deafness
- ◯ Chronic lung disease/bronchopulmonary dysplasia (preterm only)
- ◯ Other ________

* list from Core outcomes in neonatology and above list

Potential Organisational Barriers to a Clinical Trial

If we were to design a trial that compared prescribed timings of stoma closure, how would you foresee the following factors influencing this in your centre?

Only answer this question if the following conditions are met:
Answer was NOT 'Other' at question '1 [A01]' (Your role) *and* Answer was NOT 'Other' at question '2 [A02]' (Description of the type of unit you work in:)

Please choose the appropriate response for each item:

	No issue	May be a problem	Likely to be a significant problem
**Ability to access neonatal operating list at short notice**	○	○	○
**Access to a ventilated neonatal bed post-operatively**	○	○	○
**Surgeon availability e.g. flexibility within job plan**	○	○	○
**Willingness of anaesthetists to facilitate the trial**	○	○	○
**Transfer of infant to the surgical centre for the closure**	○	○	○
**Willingness of you and your colleagues to facilitate the trial e.g. sharing cases**	○	○	○

Ducey J, *et al. Arch Dis Child Fetal Neonatal Ed* 2022; 107:448–450. doi: 10.1136/archdischild-2021-322040

Please indicate if there are other likely barriers to running the trial at your unit.

Only answer this question if the following conditions are met:
Answer was NOT 'Other' at question '1 [A01]' (Your role) *and* Answer was NOT 'Other' at question '2 [A02]' (Description of the type of unit you work in:)

Please write your answer here:

In your unit, from when a decision to close a stoma is made, how long on average does it take to do this, taking into acount factors such as waiting for theatre space?

Only answer this question if the following conditions are met:
Answer was NOT 'Other' at question '1 [A01]' (Your role) *and* Answer was NOT 'Other' at question '2 [A02]' (Description of the type of unit you work in:)

Please write your answer here:

weeks

Would you be interested in attending a final consensus meeting at the end of the ToSCiN study?

Only answer this question if the following conditions are met:
Answer was NOT 'Other' at question '1 [A01]' (Your role) *and* Answer was NOT 'Other' at question '2 [A02]' (Description of the type of unit you work in:)

Please choose **only one** of the following:

- ◯ Yes
- ◯ No

Ducey J, *et al. Arch Dis Child Fetal Neonatal Ed* 2022; 107:448–450. doi: 10.1136/archdischild-2021-322040

If you would like to receive feedback about the results of ToSCiN, please provide your details below:

Only answer this question if the following conditions are met:
Answer was NOT 'Other' at question '1 [A01]' (Your role) *and* Answer was NOT 'Other' at question '2 [A02]' (Description of the type of unit you work in:)

Thank you for completing this survey.

We will provide feedback of the results at the end of the ToSCiN study.

29/09/2020 – 17:30

Submit your survey.
Thank you for completing this survey.

Ducey J, *et al. Arch Dis Child Fetal Neonatal Ed* 2022; 107:448–450. doi: 10.1136/archdischild-2021-322040
